# Physiotherapy Rehabilitation Following Lower Extremity Split Skin Grafting in Necrotizing Fasciitis

**DOI:** 10.7759/cureus.46295

**Published:** 2023-09-30

**Authors:** Vaishnavi Hatwar, Pratik Phansopkar

**Affiliations:** 1 Physiotherapy, Ravi Nair Physiotherapy College, Datta Meghe Institute of Medical Sciences, Wardha, IND; 2 Musculoskeletal Physiotherapy, Ravi Nair Physiotherapy College, Datta Meghe Institute of Medical Sciences, Wardha, IND

**Keywords:** physiotherapy intervention, rehabilitation, physiotherapy management, skin grafting, necrotizing fasciitis

## Abstract

Necrotizing fasciitis is defined as a highly progressive microbial infection. The diagnosis of necrotizing fasciitis is mostly based on clinical signs, and early diagnosis is key. There are several difficulties that patients confront following surgery. Physical therapy helps to regain the functional range of motion, enhance muscle strength, reduce stiffness and make the patient self-independent. We presented a case of a 54-year-old male with presenting complaints of pain over right lower limb while doing movements and difficulty in performing daily activities. The patient was diagnosed with necrotizing fasciitis of the right leg which was managed surgically by a skin-split graft but later the patient developed post-operative stiffness and pain for which physiotherapy was advised. A goal-oriented tailored rehabilitation approach was planned for the patient for four weeks. The patient and his family were informed about their present condition. Positioning was given to reduce edema. Wound healing was enhanced by the use of ultrasound and low-level laser therapy. In order to mobilize scar myofascial release technique and massage were applied. To increase functional range and strength, a mobility and strengthening exercise was performed. To make the patient functionally independent balance and gait exercise was given. The outcome measures included a numerical pain rating scale (NPRS), range of motion, manual must testing, and lower extremity functional scale. There was an improvement in all the outcome measures taken. The treatment protocol proved to be beneficial for the patient.

## Introduction

Necrotizing fasciitis is a rapidly progressing microbial infection that can damage any soft tissue layer (dermis, subcutaneous tissue, superficial fascia, deep fascia, and muscle) separately or in any combination [1]. The diagnosis of necrotizing fasciitis is mostly based on clinical signs, and early diagnosis is key. It rarely includes muscles and usually occurs in areas with loose tissue. The majority of cases spread quickly to local tissue. Patients typically develop sepsis and die of septic shock if they are not properly handled; later stages of the disease are usually followed by multiple organ failure [2]. When a patient has a history of local trauma, local inflammation, fever, or hypothermia, necrotizing fasciitis should be suspected, and a differential diagnosis between necrotizing fasciitis and cellulitis should be made [3]. This condition, also known as "flesh-eating disease," commonly affects the limbs, usually, unilaterally, however occasional examples of bilateral and multifocal involvement have been recorded in the literature [4]. Microbiologically, necrotizing fasciitis is classified into Type I (polymicrobial) and Type II (monomicrobial). Mono-microbial infections are less prevalent, often affecting the limbs of healthy individuals with no accompanying co-morbidities [5].

A quick diagnosis followed by several surgical debridements is key to a positive outcome. In this case, a surgical debridement along with intravenous benzylpenicillin therapy and split skin grafting was done. Because skin grafting is a surgical process, it has several risks, including bleeding, respiratory issues, infection, adverse drug reactions, and so on [6]. Lower limb skin grafts fail often. BMI, immunosuppressant use, and peripheral vascular disease (PVD) appear to be substantial risk factors for graft failure. These criteria must be considered during preoperative evaluation to identify individuals who are at a higher risk of postoperative problems [7].

Furthermore, graft failure can result in infection, edema, and decreased blood supply to the injured area; the most typically observed symptom is the development of a hematoma. Any physical activity, such as stretching or compressing the graft, might have negative consequences. The graft should be monitored for at least 15 to 30 days to avoid such complications. Physical therapy combined with ultrasonic massage can help individuals suffering from developing deformities and therefore retain skin integrity permanently [6]. Postoperatively patient encountered many difficulties. Physical therapy helps to regain the functional range of motion, enhance wound healing and muscle strength, mobilize scar, reduce stiffness and make the patient self-independent. A goal-oriented tailored rehabilitation approach is very important in a condition like this. The goal of this study is to prevent complications and make the patient functionally independent. It provided awareness about the post-surgical importance of physiotherapy. 

## Case presentation

Patient information

A 54-year-old male welder by occupation with right-hand dominance came to the musculoskeletal OPD with the complaint of pain while doing movement and difficulty in performing activities for two and a half months. As per the history given by the patient, the patient had itching on the left leg, and on September 14 patient noticed two blisters around the left knee joint following which the patient developed swelling and erythema. He then visited the government hospital of Yavatmal and their medicine was prescribed. He didn’t get relief from the medication so he visited a private hospital where the doctor advised some investigation on blood culture; Pseudomonas aeruginosa was present and he was diagnosed with necrotizing fasciitis. The first operation was done on September 19 when he underwent debridement of the right lower limb. The operation was not so eventful so he came to the Acharya Vinoba Bhave Rural Hospital (AVBRH) for further management. In the surgery department, the patient was managed. A skin split graft surgery was done on October 1. The graft was taken from the anterior, medial, and lateral aspects of the left thigh using Watson modification of humbys knife.

Clinical assessment

**Figure 1:**
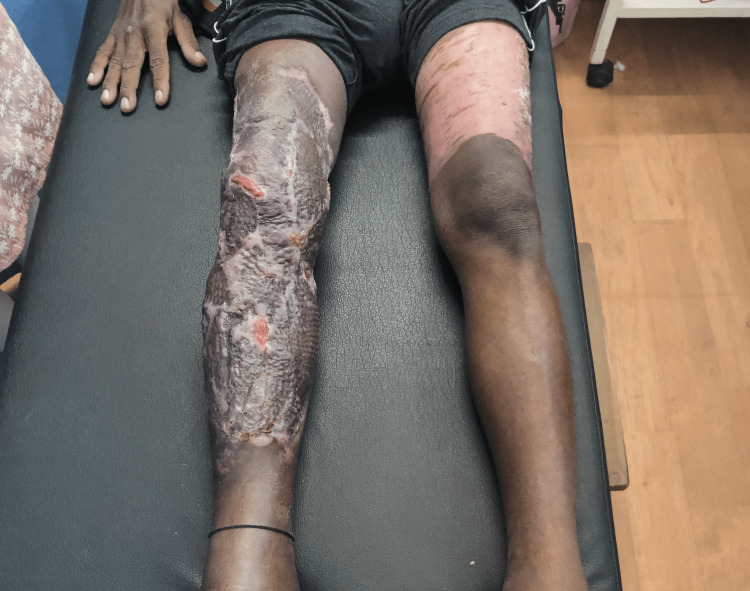
Right leg showing graft site and left leg showing donor site

Clinical evaluation was done after obtaining the patient's consent. The patient was seen supine lying with his right leg elevated on a pillow. The patient was alert, attentive, cooperative, and well-oriented to time place, and person. His BMI was 26 kg/m2, his pulse rate was 82 bpm, and he was afebrile on general examination. Pitting edema was evident in the right lower limb up to the ankle. On the numerical pain rating scale (NPRS), pain was 7/10 on movement and activity, which was gradual in onset and progressive in nature. On observation, partially healed skin grafting was seen on the right leg and a scar was present on the left thigh as shown in Figure 1. On palpation, grade 2 pitting edema and warmth were present up to the ankle joint in the right lower limb. On scar examination, the length and width of the right side scar were 53cm and 29cm and of the left side 30cm and 40cm. On examination, sensations and reflexes were intact and hyperesthesia was present on the left thigh. There was no limb length discrepancy present. Range of motion was taken of bilateral lower extremity both actively and passively which is given in Table 1 and manual muscle wasting in Table 2.

**Table 1:** Pre-treatment range of motion (ROM)

Joint	Left Active	Left Passive	Right Active	Right Passive
Hip				
Flexion	0-110°	0-120°	0-90°	0- 95°
Extension	0-25°	0-30°	0-15°	0-20°
Abduction	0-45°	0-45°	0-35°	0-40°
Adduction	0-35°	0-35°	0- 25°	0-30°
Knee				
Flexion	0-130°	0-130°	0-90°	0-90°
Ankle				
Plantar Flexion	0-45°	0-45°	0-45°	0-45°
Doris Flexion	0-20°	0-20°	0-10°	0-10°
Inversion	0-30°	0-35°	0-10°	0-10°
Eversion	0-20°	0-25°	0-10°	0-10°

**Table 2:** Pre-treatment manual muscle testing

Hip	Right	Left
Flexor	3/5	4/5
Extensor	3/5	4/5
Abductor	3/5	4/5
Adductor	3/5	4/5
Knee		
Flexor	3/5	4/5
Extensor	3/5	4/5
Ankle		
Plantar flexor	4/5	5/5
Dorsi flexor	4/5	5/5

Investigations

Blood culture was positive for Pseudomonas aeruginosa.

Therapeutic management

**Figure 2:**
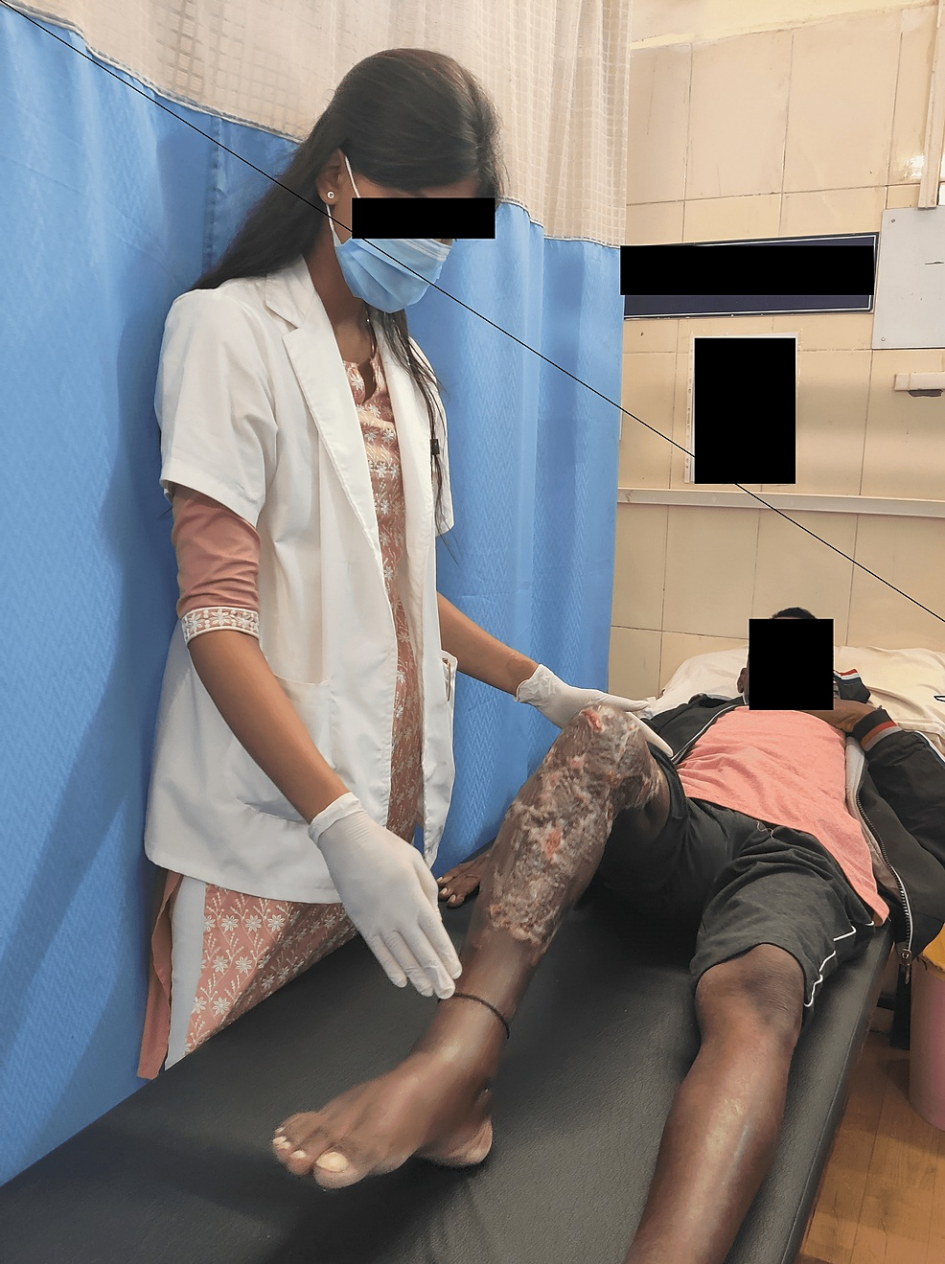
The patient performing heel slides.

**Figure 3:**
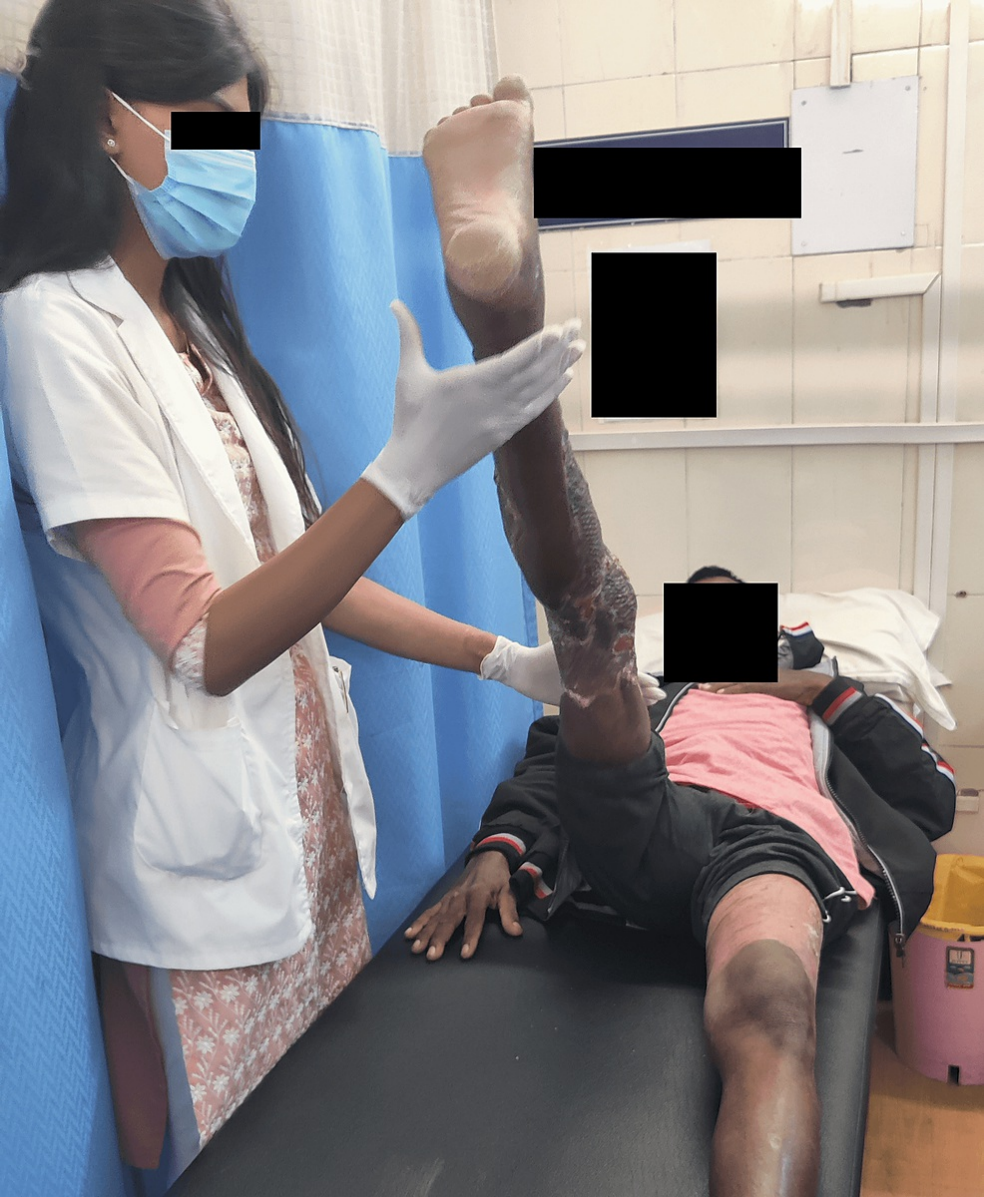
The patient performing active assisted movements.

The rehabilitation protocol was started from the day patient visited the musculoskeletal department OPD. A tailor-made protocol was given for four weeks (Table 3). Exercise protocol was performed by patient as shown in Figures 2, 3.

**Table 3:** Physiotherapy Intervention

Problems faced by patients	Goals	Intervention
Stress, anxiety, and depression	To educate patient	Counseled the patient about his skin conditions and the need for physiotherapy, as well as how physical therapy will assist him to improve his condition. The patient was motivated to re-establish themselves in their social and occupational activities as quickly as possible, and their family member was encouraged to support this behavior as well.
Edema below the ankle	To reduce edema	The right leg was elevated with two pillows.
Prevent deep vein thrombosis (DVT), contractures, infection, pressure sores	To prevent secondary complications such as DVT, and contracture, avoid damage to new skin grafts and avoid pressure sores	Ankle toe movements (10 reps for 3 sets), positioning and splinting to prevent contracture, and passive movements (10 reps for 3 sets)
Delay in wound healing	To enhance wound healing	Ultrasound at an intensity of 0.8 w/cm2 for 8 minutes in 1MHz for 7 days, Low-level laser therapy was applied to the skin graft donor site for 7 minutes for 5 days.
Scar around graft	To mobilize soft tissue around the scar	Massage (3-way massage which is rounded up and down, and side to side), Myofascial release with the thumb performed 3-4 times per day for 5-10 mins. It was taught to the patient relative so that they can perform it at home.
Reduced range of motion, stiffness	To improve the range of motion and to prevent stiffness	Active range of motion in pain-free range progression to full range of motion, active assisted range of motion, and passive range of motion (10 reps for 3 sets) and Maitland mobilization grade 2 and 3 for tibiofemoral joint in A/P and P/A directions on the affected side were given (10 reps for 3 sets). Stretching exercises for the hamstring and Achilles tendon were given to reduce stiffness (10 reps for 3 sets).
Muscle weakness	To improve and maintain the muscle strength	Static hamstring, static quadriceps, static abdominals, static glutes, dynamic quadriceps (each for 10 reps for 3 sets), strengthening initiation with half kg weight progression to 1 kg and then TheraBand with 5-sec hold (10 reps for 3 sets).
Difficulty in walking	To make the patient self-ambulatory	Bedside sitting, sit to stand, stand without support
Difficulty in maintaining balance	To improve the balance	Step up step down, balancing on wobble board with stride standing, step standing, and single leg standing.
Abnormal gait pattern	To improve the gait style of patient	Walking with a walker, walking in the parallel bar, tandem walking, side walking, and walking on marked points.

Follow-up and outcomes 

**Figure 4:**
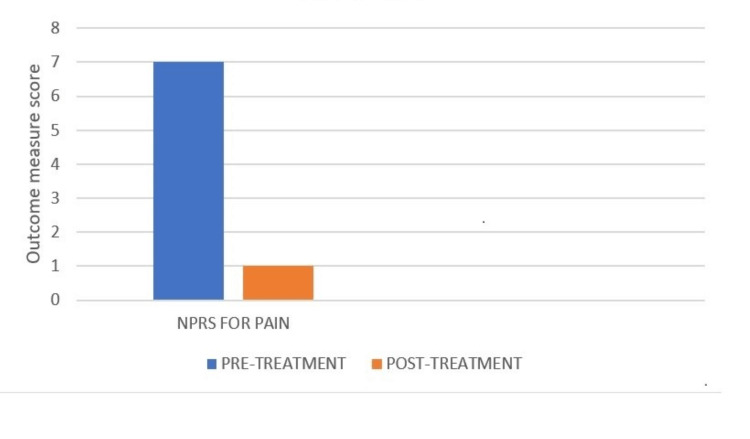
The Numerical Pain Rating Scale (NPRS) NPRS is a one-dimensional pain intensity scale. A 10-point scale is used, with zero representing "no pain" and 10 representing "worse pain".

**Figure 5:**
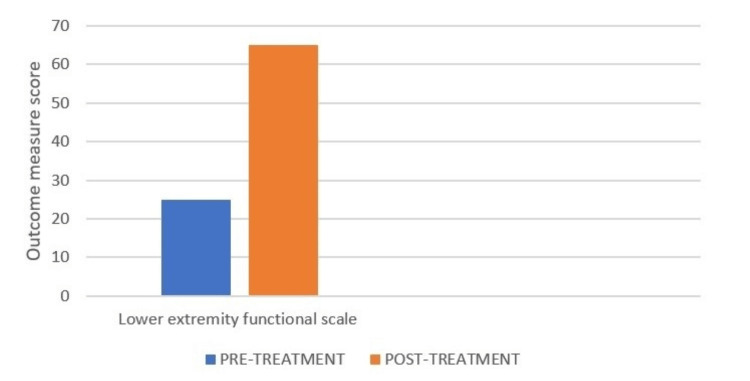
Lower extremity functional scale (LEFS) LEFS is a valid patient-rated outcome measure for the measurement of lower extremity function. The total score of LEFS is 80. Pre-treatment LEFS score was 25 and post-treatment LEFS score was 65.

A four-week follow-up was performed. The patient was able to walk without assistance and do basic daily tasks. The patient was encouraged to finish physical therapy. In addition, the patient was given information on home exercise regimens and was advised to continue strength training. On the day of the examination, the score on the NPRS was 7/10. Following physical treatment, improvements in NPRS were estimated to be 1/10 (Figure 4). The patient's increased range of motion was a sign of progress (Table 4). The strength of the lower extremities was also improved (Table 5). Lower extremity functional scale also showed improvement as seen in Figure 5. Improvements were noted as the patient progressed through the physical therapy.

**Table 4:** Post-treatment range of motion (ROM) The ROM of the considered joint was measured by goniometry.

Joint	Left Active	Left Passive	Right Active	Right Passive
Hip				
Flexion	0-120°	0-120°	0-110°	0-115°
Extension	0-30°	0-30°	0-15°	0-20°
Abduction	0-45°	0-45°	0-40°	0-45°
Adduction	0-35°	0-35°	0-30°	0-35°
Knee				
Flexion	0-130°	0-130°	0-120°	0-125°
Ankle				
Plantar Flexion	0-45°	0-45°	0-45°	0-45°
Dorsi Flexion	0-20°	0-20°	0-20°	0-20°
Inversion	0-30°	0-35°	0-30°	0-35°
Eversion	0-20°	0-25°	0-20°	0-25°

**Table 5:** Post-treatment manual muscle testing (MMT) Muscle testing was performed and scoring was based on MMT.

Hip	Right	Left
Flexor	4/5	5/5
Extensor	4/5	5/5
Abductor	4/5	5/5
Adductor	4/5	5/5
Knee		
Flexor	4/5	5/5
Extensor	4/5	5/5
Ankle		
Plantar flexor	5/5	5/5
Dorisi flexor	5/5	5/5

## Discussion

In this case study, we have discussed the case of a 54-year-old male with a history of necrotizing fasciitis who underwent a surgical procedure skin graft of his right leg, and the graft was taken from the thigh of the left side. Later, came to the musculoskeletal department with complaints of pain while doing movements and difficulty in doing movements. The goal of physiotherapy rehabilitation is to reduce pain, prevent secondary complications, and make the patient functionally independent as early as possible. Prolonged bed rest improves neither graft take nor overall patient outcomes and raises the risk of venous thromboembolism (VTE), as well as limiting movement and raising the risk of deconditioning [8]. Early mobilization has shown improvement in patients who underwent skin graft procedures [9].

Complications after skin graft are infection, problems with blood circulation, contracture, loss of skin sensation and increased sensitivity to pain, chronic pain, and scar tissue building up around tissue. Physical therapy helps to minimize the chances of scar tissue interfering with your walking. The physiotherapy was started by educating the patient about the condition, reducing the edema, improving the range of motion and improving and maintaining the strength of the patient, and making the patient ambulatory. Individuals must continue a regular exercise regimen, which will assist in stretching scar tissue while also boosting exercise tolerance and maintaining a positive mental state. 

The patient presented with edema over the left leg so we prescribed him the positioning. We asked the patient to keep two pillows below the limb while lying on a plinth. The effects of ultrasonic treatment on skin-grafted patients were found to be substantial. The study demonstrated the efficacy of ultrasonic therapy in generating functional and morphological changes at the cell level, resulting in the development of new blood vessels and the reconnection of the entire thickness of the graft [6]. Then we started with ankle-toe movements, heel slides, and passive movements to prevent secondary complications such as deep vein thrombosis (DVT), peripheral vascular diseases, pressure sore, etc. To prevent post-operative respiratory complication breathing exercise was given. Splinting was employed to prevent contracture. Avoiding knee flexion contracture can be accomplished by keeping the legs extended when resting or sitting, as well as by using knee extension splints. Splinting aids in the maintenance of anti-contracture posture, especially in patients who are in a lot of pain [10]. Then active, active assisted, and passive range of motion exercise was given to improve the functional range of the patient. Maitland grade 2 and 3 mobilizations were given for the tibiofemoral joint. Stretching exercises for the hamstring and Achilles tendon were given to reduce stiffness. For improving the strength of the muscles initiated static isometrics and then progression to dynamic for the hamstring, quadriceps, glutes, and abdominals. Massaging can break the collagen bundles present in the graft and form a scar. Myofascial release (MFR) is given to mobilize the soft tissue and to reduce dysfunctions in soft tissue that cause pain and limit motion in the left leg. Sensory impairment and changes in cutaneous sensation are common in scars. Regular massage and touching of the scars help with the desensitization of hyper-sensitive scars [11]. 

When we used this approach on our patient, it resulted in a good recovery and was also therapeutic. We encountered limitations such as discomfort, restricted mobility, and delayed wound healing. Individuals should begin physiotherapy as soon as possible to avoid future difficulties.

## Conclusions

Physiotherapy plays an important role in preventing complications and improving the functional ability of patients. The given physiotherapy protocol for the patient who underwent surgery with a split skin graft proved to be beneficial in enhancing the patient's functional independence in this case report. There was an improvement in all the outcome measures taken. The treatment protocol proved to be beneficial for the patient.
